# A larger TatBC complex associates with TatA clusters for transport of folded proteins across the bacterial cytoplasmic membrane

**DOI:** 10.1038/s41598-024-64547-x

**Published:** 2024-06-14

**Authors:** Max-Hinrich Werner, Denise Mehner-Breitfeld, Thomas Brüser

**Affiliations:** Institute of Microbiology, Leibniz Universität Hannover, Herrenhäuser Straße 2, 30419 Hannover, Germany

**Keywords:** Microbiology, Cellular microbiology, Membrane proteins

## Abstract

The twin-arginine translocation (Tat) system transports folded proteins across energized biological membranes in bacteria, plastids, and plant mitochondria. In *Escherichia coli*, the three membrane proteins TatA, TatB and TatC associate to enable Tat transport. While TatB and TatC together form complexes that bind Tat-dependently transported proteins, the TatA component is responsible for the permeabilization of the membrane during transport. With wild type Tat systems, the TatB- and TatC-containing Tat complexes TC1 and TC2 can be differentiated. Their TatA content has not been resolved, nor could they be assigned to any step of the translocation mechanism. It is therefore a key question of current Tat research to understand how TatA associates with Tat systems during transport. By analyzing affinity-purified Tat complexes with mutations in TatC that selectively enrich either TC1 or TC2, we now for the first time demonstrate that both Tat complexes associate with TatA, but the larger TC2 recruits significantly more TatA than the smaller TC1. Most TatA co-purified as multimeric clusters. Using site-specific photo cross-linking, we could detect TatA–TatC interactions only near TatC transmembrane helices 5 and 6. Substrate-binding did not change the interacting positions but affected the stability of the interaction, pointing to a substrate-induced conformational transition. Together, our findings indicate that TatA clusters associate with TatBC without being integrated into the complex by major rearrangements. The increased TatA affinity of the larger Tat complex TC2 suggests that functional assembly is advanced in this complex.

## Introduction

Tat systems are translocons that serve to transport folded proteins across biological membranes^1^. In *Escherichia coli*, the Tat system is functional with three components, TatA, TatB, and TatC. There are two TatA paralogs in *E. coli*, TatA and TatE, which have overlapping functions^2^. Structures of TatA, TatB, and TatC have been solved by NMR spectroscopy or crystallography^3–7^, but the complexes formed by the interacting components remained unclear. Two such complexes, Tat complexes TC1 and TC2 have been detected by blue-native polyacrylamide gel electrophoresis (BN-PAGE), with TC2 migrating at a higher apparent molecular mass than TC1^8,9^. Both complexes can bind Tat substrates^10^. A third complex, TC3, which is slightly larger than TC2, has been identified with TatC mutations that apparently delayed resetting of the translocon after transport^8^. It is highly important to understand whether the known Tat complexes represent certain assembly intermediates, and/or whether they represent certain states of the translocation pathway. TatB and TatC protomers are believed to exist in equal numbers in Tat translocons^11^, but it is known that TatA can in principle occupy TatB binding sites at TatC, and an exchange of TatB by TatA is enhanced by active transport^12^. TatA and TatB are structurally highly similar and belong to the same protein family^13^, and there exist two-component Tat systems in which one TatA family protein serves both functions^14,15^. In three-component Tat systems, such as in *E. coli*, most TatB is found to be tightly associated with TatC, whereas most TatA is found dissociated from TatC-containing complexes after cell disruption and solubilization^9,16^. TatA is engaged by the system during transport^17,18^. It has been shown that TatA can destabilize the cytoplasmic membrane^19,20^, and MD simulations gave mechanistic explanations for this^21^, supporting the idea that associations of multiple TatA protomers at active translocons permit a membrane passage by a membrane-weakening and pulling mechanism^22^. Membrane-destabilization by antimicrobial peptides has been shown to stimulate Tat transport, which further agrees with this model^23^. However, also mechanisms have been proposed that assume a TatA-dependent formation of an aqueous hole by iris-like openings either outside or inside of TatBC-containing complexes, but it is unclear how such structures could be formed^24^.

In this study, we address the question how TatA associates with the translocon. Our data indicate that the larger TC2 represents a more advanced assembly state with higher affinity to TatA than found for TC1. Further analyses indicate that TatA clusters are laterally recruited to unaltered TatC binding sites, which likely is of key importance for the translocation mechanism.

## Results

### Mutations in TatC can selectively stabilize TC1 or TC2

**Figure 1:**
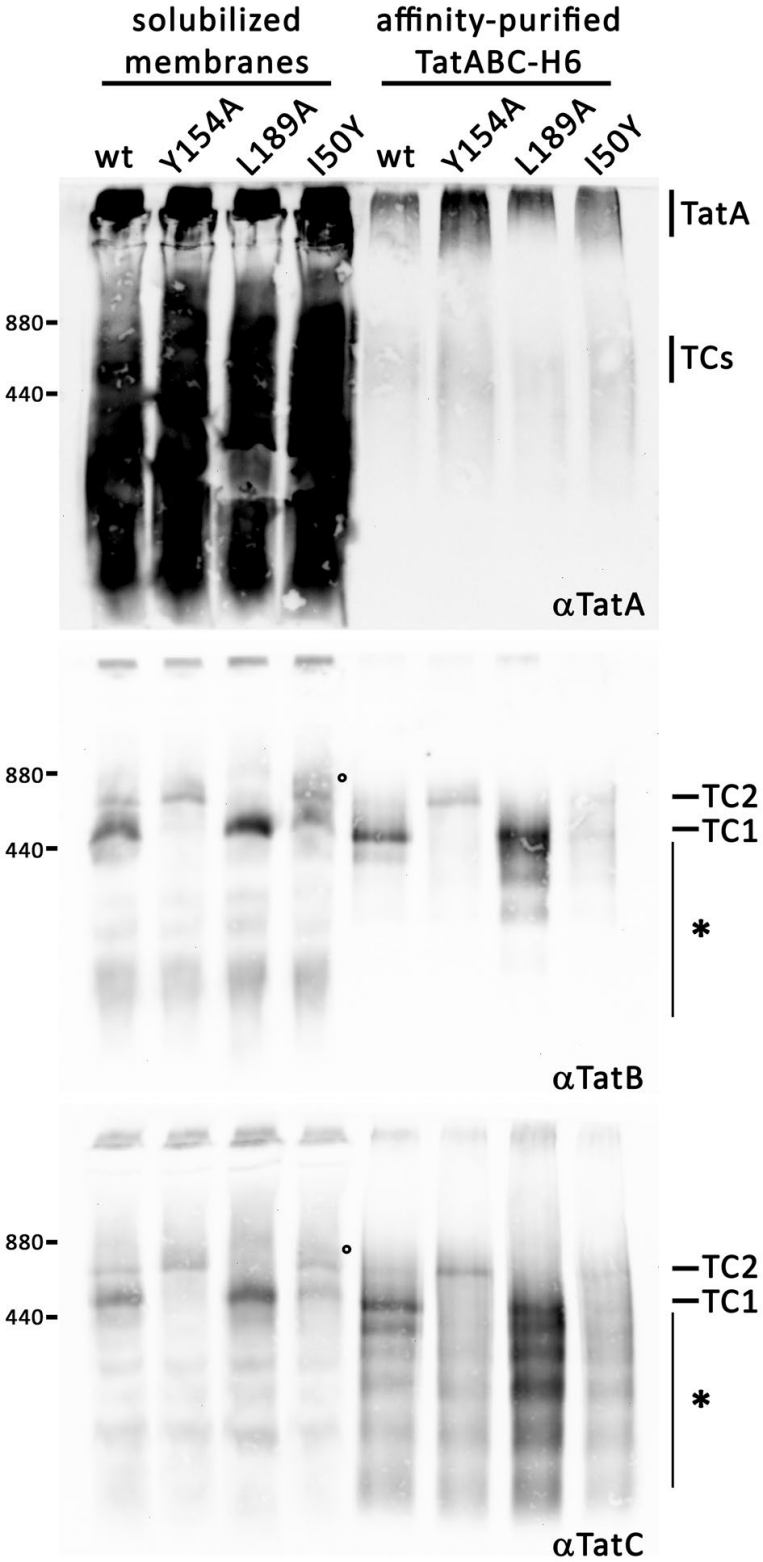
BN-PAGE analyses of TatABC components with mutations in TatC that selectively stabilize TC1 or TC2. Samples of crude solubilized membranes (left four lanes) and affinity chromatography-purified samples (right four lanes) with the indicated Tat systems (wild type or indicated TatC mutations) were analyzed by BN-PAGE. Blots were developed using antiserum against TatA (upper blot), TatB (middle blot) or TatC (lower blot). Positions of Tat complexes (TC1 and TC2) are indicated on the right of TatB and TatC blots. TC1 and TC2 correspond to the complexes that were previously termed 440 kDa and 580 kDa complexes, which have been renamed as the migration behavior depends on the lot of digitonin used^8^. °Weak TC3 band; *Region with signals from disassembly products, as identified in previous studies^10^. In case of the TatA blot, the detected high molecular weight co-purified TatA assemblies are indicated (TatA), as well as TatA signals in the region of TatBC-containing complexes (TCs). Note that TatA associates to a wide range of homooligomers that stain whole lanes without purification (solubilized membranes). Positions of size markers are indicated on the left.

Although digitonin-solubilized Tat system components are known for long to assemble to various complexes that can be distinguished by BN-PAGE analyses^8,25^, the analysis of the relative content of TatA, TatB, and TatC in specific complexes has been hampered by the fact that TC1 and TC2 so far could not be separated for biochemical analyses. Many mutated Tat systems have been analyzed in the past, and several amino acid exchanges, all in TatC, have been identified that shifted the Tat complexes either to TC1 or to TC2. Especially TatC(Y154A) has been described to shift the TatBC-containing complexes to TC2, and TatC(L189A) shifted the complexes to TC1^10^. Also TatC(I50Y) was relevant in this context, as this mutation resulted in similar abundances of TC1, TC2, and TC3^8^. TatC(Y154A), TatC(L189A), and TatC(I50Y) are active, as previously shown^8,10^. We attempted to use these mutated Tat systems for a biochemical analysis of the relative Tat component contents in the Tat complexes. As in most previous studies, we used a recombinant constitutive expression system, which is fully functional and which gives rise to the same two Tat complexes that can be detected at wild type expression level^9^. TatA is highly abundant in the membranes, and homooligomeric TatA associations of a wide range of sizes are detected as a dense ladder of bands in BN-PAGE that reaches from low to very high molecular weight, precluding any TatA content assessments of non-purified Tat complexes. To learn about the relative TatA content in the Tat complexes that can be differentiated by BN PAGE, we therefore first needed to separate free TatA from the Tat complexes by purification of the mutated Tat complexes (Fig. 1). Purification was done by immobilized metal affinity chromatography (IMAC) of TatABC complexes using a hexahistidine-tag at the C-terminus of TatC. TC2 remained intact in preparations of TatABC(Y154A), with no formation of TC1 during the purification, and TC1 remained intact in preparations of TatABC(L189A), likewise not showing any TC2 after affinity chromatography. The wild type showed some depletion of TC2 during purification, indicating that TC2 is less stable than TC1, and TC2 can convert to TC1 if TC2 is not stabilized by mutations such as TatC(Y154A). Note that TC2 usually is less abundant than TC1, and harsher solubilization protocols reduce the TC2 content, permitting the detection of TC2 only when stabilizing mutations are analyzed^26^. In case of TatABC(I50Y), the complexes TC1 and TC2 were still detectable after affinity-purification in comparable amounts without significant decrease of TC2 abundance, whereas TC3—which was already hardly seen before purification—was not detectable anymore. The TatA detections showed that free TatA was efficiently removed by the affinity chromatography, and weak TatA signals remained detectable in the size range of TC1 and TC2 after purification. These signals appeared somewhat stronger in case of TC2-stabilization by mutations such as TatC(Y154A) and TatABC(I50Y), indicating that possibly more TatA was bound to the corresponding Tat complexes. In TatA blots, TatABC complexes were detected as more diffuse bands, possibly due to variable numbers of TatA that remained bound to a small portion of the TatBC core. Interestingly, most TatA was detected at the upper edge of the gel, indicating the co-purification of very large TatA assemblies, and these TatA signals were strongest in case of TatABC(Y154A) and also enhanced relative to the wild type in case of TatABC(I50Y), which are both mutated Tat systems with stabilized TC2 relative to the wild type. In summary, the stabilization of TC2 by TatC mutations Y154A or I50Y increased the amount of co-purified TatA, which could be detected by BN-PAGE, and while a small portion of this TatA migrated with TatBC complexes in the known size range of these complexes, most TatA co-purified as largely homooligomeric/homomultimeric TatA assemblies.

### TC2 binds significantly more TatA than TC1

**Figure 2:**
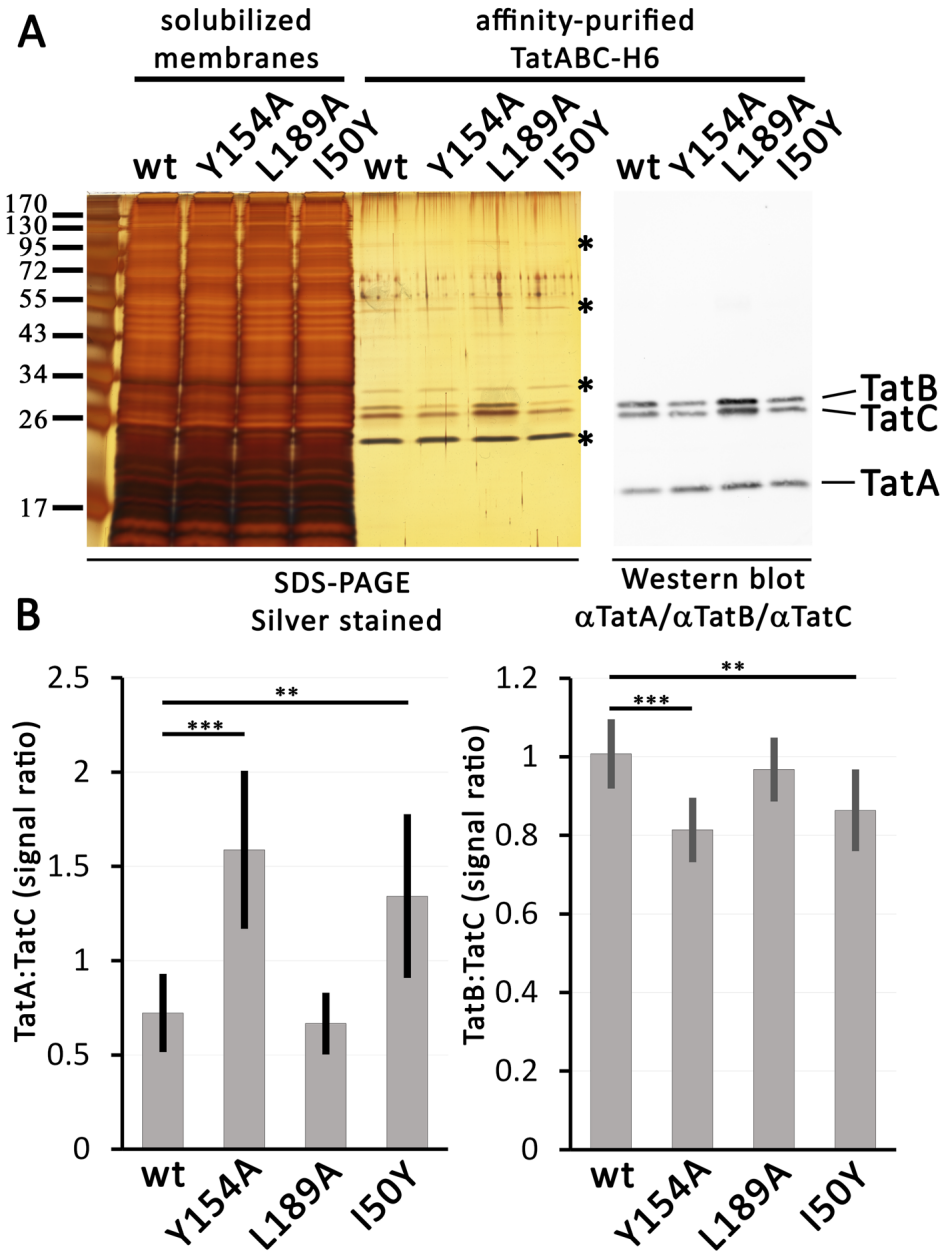
TC2-stabilization correlates with increased TatA amounts. (**A**) Analyses of wild type (WT) and the TatC-mutated Tat systems TatABC(Y154A), TatABC(L189A), and TatABC(I50Y). SDS-PAGE/silver stain analysis of samples before and after affinity purification, and Western-blot analysis of the purified samples, as indicated, using a mix of antisera for the detection of TatABC. Asterisks (*) indicate positions of Tat-unrelated unknown bands. (**B**) Quantification of the TatA:TatC and TatB:TatC signal ratio in purified samples of the indicated Tat systems (error bars indicate standard deviations calculated from eight replicates; *t*-test significance results are indicated above the corresponding columns: ***p ≤ 0.001; **p ≤ 0.01).

As these experiments had shown that purified complexes were suitable for the analysis of the relative content of Tat system components purified with TC1 or TC2, and as the BN-PAGE data suggested an increased amount of TatA in association with TC2, we analyzed the changes in TatA:TatC and TatB:TatC ratios caused by the mutations that either stabilized TC1 or TC2, using SDS-PAGE/Western blots, developed by mixtures of antibodies directed against TatA, TatB, and TatC (Fig. 2). Proteins separated by SDS-PAGE were also silver-stained, and the comparison of crude solubilized membranes with the samples after affinity-purification demonstrated a high degree of purification, with some contaminating proteins still detectable (Fig. 2A). Most contaminating bands were not Tat-dependent as a mock purification without tagged Tat systems gave the same bands (Supplementary Fig. S2). However, we could Tat-dependently co-purify PspA (Supplementary Fig. S2), a membrane-stress response protein that interacts with the membrane-stress-inducing TatA membrane anchor in *E. coli*^27^, and that also has shown to be involved in regulation of the *tatABC* operon in *Salmonella enterica*^28^. LiaH, a PspA homolog in *B. subtilis*, is known to interact with TatAyCy^29^, the general Tat system in this species, and our co-purification with tagged TatC now shows that PspA similarly also interacts with the TatABC system in *E. coli*. TatA could not be stained by this method, but the components TatB and TatC were nicely detectable. All three components were readily detected by Western-blot analysis, and the specificity of the TatA detection was confirmed by control experiments (Supplementary Fig. S1). The band intensities were not the same for the four analyzed strains, and a quantification showed that in case of TatABC(Y154A) more TatA was bound, whereas in case of TatABC(L189A) the intensities did not differ significantly from the wild type Tat system (Fig. 2B, left diagram). As the wild type Tat system had lost almost all TC2 during purification, and only TC1 was left—just like in case of the TatABC(L189A) system—these data indicated that TC2-preparations must have contained more TatA than TC1. Also, in case of the TatABC(I50Y) system, the TatA abundance was significantly increased, which agrees with the finding that TC2 of this Tat system resisted solubilization and purification, and therefore TC2 led to an increased amount of TatA in this preparation. Notably, there was also a small effect on the TatB:TatC ratio (Fig. 2B, right diagram). It therefore might be that part of the TatB can be substituted by TatA in TC2, which is the larger Tat complex that apparently has an enhanced affinity to TatA. Such a substitution has been proposed, based on disulfide cross-linking data^12^.

### TatA cross-links to the short periplasmic loop between transmembrane helices V and VI of TatC, which is also a TatC/TatC contact site

To analyze the TatA interaction with TatC further, and to be able to study potential differences of these interactions in TC1 and TC2, we placed the photo-activatable cross-linker amino acid *p*-benzoyl-phenylalanine (Bpa) at previously reported TatA interaction sites^12,30,31^, and screened these sites for TatA occupancy by photo cross-linking. In principle, there are three binding sites reported for TatA at TatC, two that have been identified in *E. coli* at a site that has been termed ‘polar cluster site’, which is positioned adjacent to transmembrane helices (TMH) V and VI^12,32^, and a third that has been found at TMH IV in the concave lateral surface of TatC^30^. TatA binding sites at the ‘polar cluster’ were examined by Bpa positioned at M205 (TMH V site), F213 (TMH VI site), and P210/D211 in the short region connecting helices V and VI. Also N139 was included in the analysis, as the corresponding position of chloroplast TatC (N203) contacts TatA most likely when TatA is bound at TatC TMH VI^30^. The possibly weaker third binding site at TMH IV, which has been identified in pea chloroplast TatC at position L231 (corresponds to *E. coli* TatC position V167)^30^, and very weakly also in *E. coli* TatC at position A160^31^, was monitored by Bpa at these positions (V167, A160), and by a position nearby that points to TMH IV (F76 at the beginning of TMH II).

**Figure 3:**
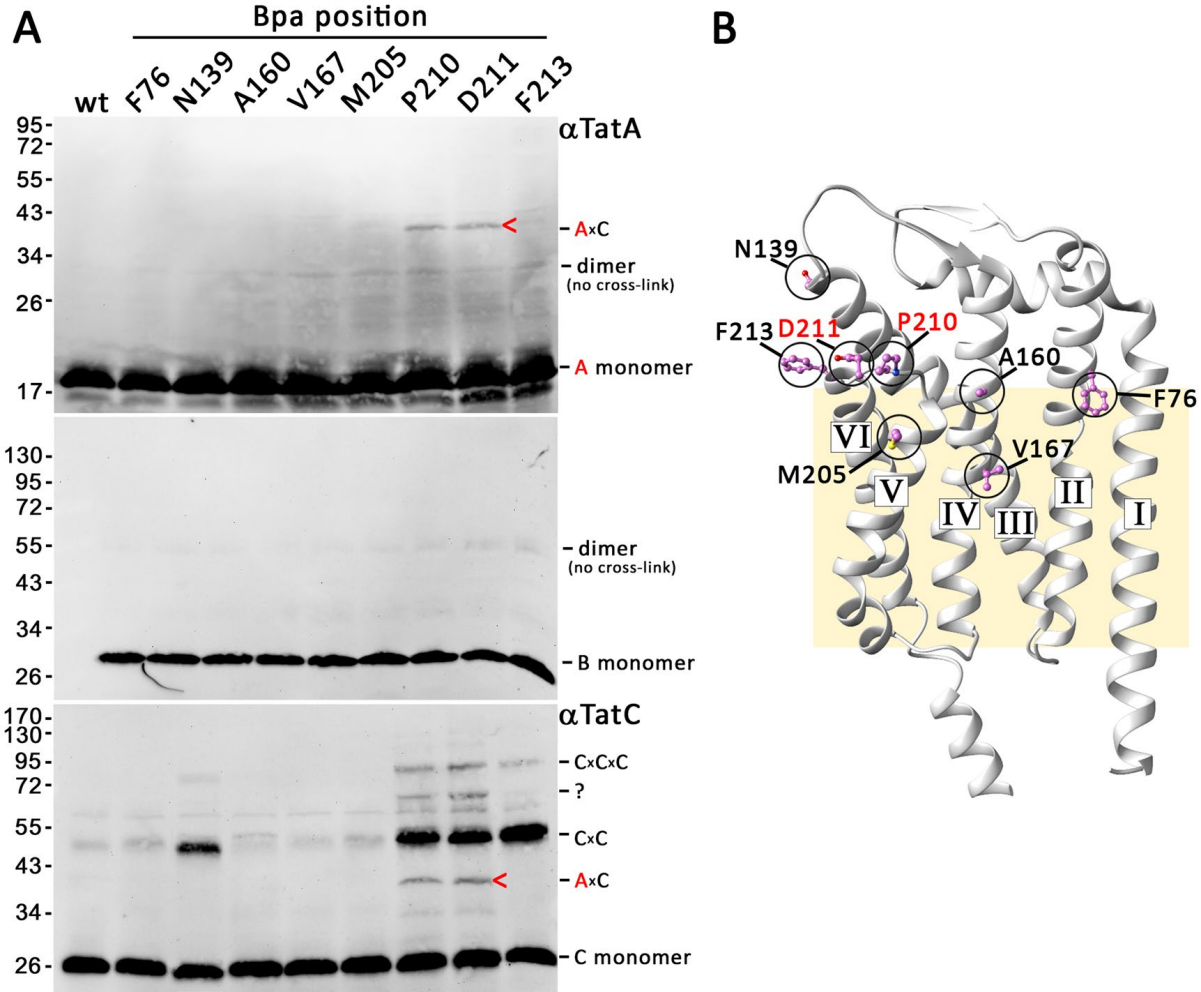
(**A**) Bpa cross-linking analysis for the detection of TatA cross-links to indicated positions in TatC. Bpa-positions cover the known TatA interaction sites. Positions of assigned bands with TatA (upper blot), TatB (middle blot), and TatC (lower blot) are indicated on the right. Molecular weight marker positions are shown on the left. Red arrowheads indicate TatA cross-links. Cross-link positions are indicated on the right. TatA × TatC cross-links are assigned based on coincident positions of the bands in TatA and TatB blots, and assignment of other cross-links is based on their apparent size. A cross-link with an unknown partner is indicated by a question mark. (**B**) Analyzed positions of Bpa exchanges in TatC. The structure was modeled by AlphaFold 2^52^.

As a result, we found that the short periplasmic loop between TMH V and VI of TatC clearly cross-linked with TatA when Bpa was positioned at P210 or D211 (Fig. 3). Both positions were also in close proximity to neighboring TatC protomers, as TatC × TatC and TatC × TatC × TatC cross-links were formed. In addition, we also detected TatC × TatC and TatC × TatC × TatC cross-links with Bpa at positions N139 and F213, but these positions did not contact TatA. We could not detect any TatB cross-link, not even to M205Bpa, which has been seen in a previous in vitro cross-linking study to contact TatB^31^ (see “Discussion”). As expected for UV-induced cross-links, all detected cross-links depended on UV-irradiation (Supplementary Fig. S3). In summary, TatA cross-links could only be obtained with TatA at the ‘polar cluster’ site with Bpa placed in the periplasmic loop between helices V and VI.

**Figure 4:**
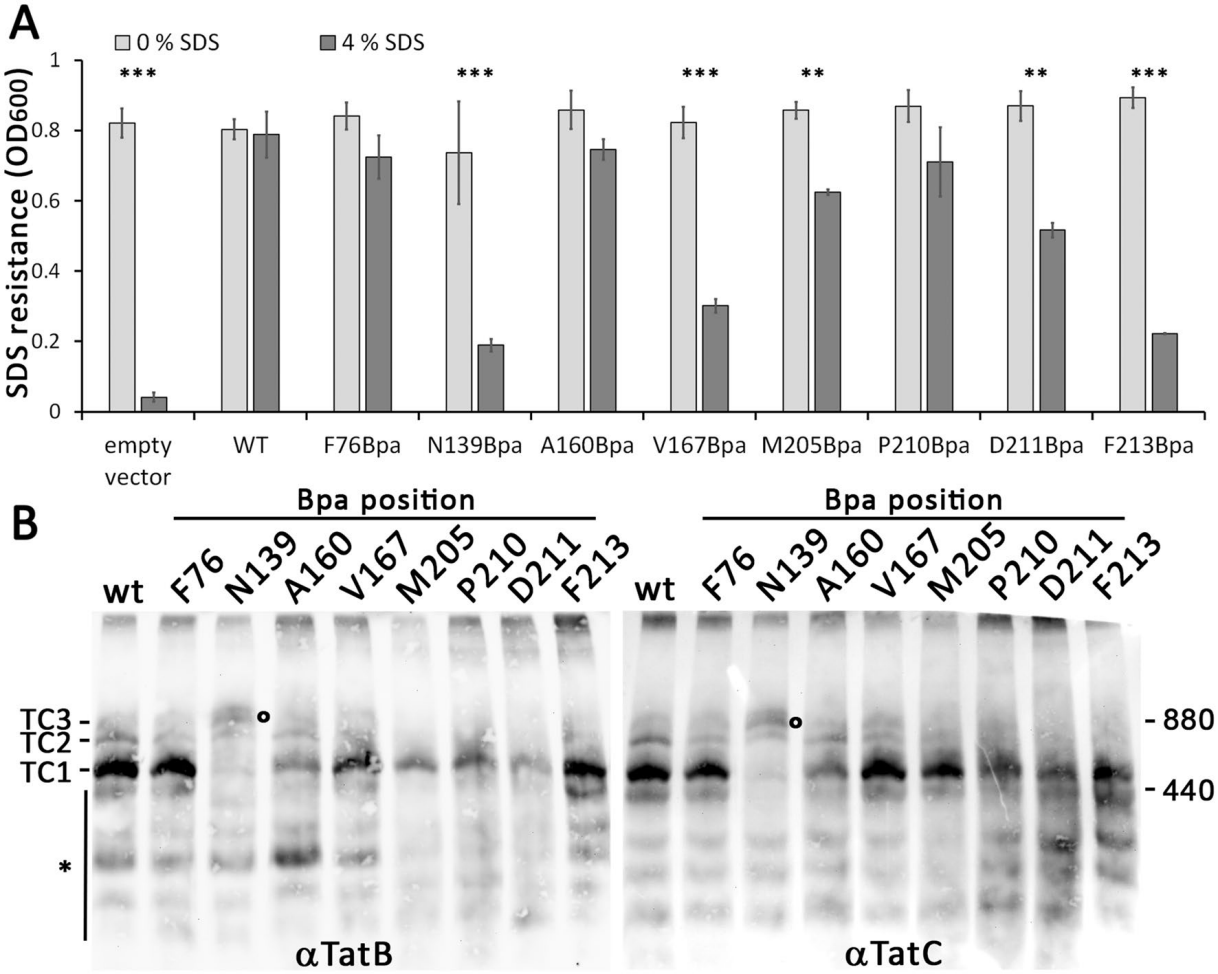
(**A**) Tat functionality of the analyzed Tat systems, as monitored by determination of SDS resistance (see “Methods”). *t*-test significance results are indicated above the corresponding columns and monitor the significance of normalized SDS sensitivity (ratio ± SDS) in comparison to the wild type strain: ***p ≤ 0.001; **p ≤ 0.01. (**B**) BN-PAGE analysis of the influence of Bpa exchanges at indicated TatC positions on Tat complex formation. The circle indicates the position of higher molecular weight complexes with TatC(N139Bpa), which hardly forms TC1 (see text for details). *Region with signals from disassembly products.

To test whether the Bpa substitutions affect transport activity of the complexes, which would be expected if important interactions are disturbed, we examined Tat functionality by determination of SDS sensitivity. In case of compromised Tat transport, the lack of Tat-dependently translocated cell wall amidases AmiA and AmiC in the bacterial periplasm affects outer membrane integrity, resulting in increased SDS sensitivity^33^. We found that TatC(N139Bpa), TatC(V167Bpa) and TatC(F213Bpa) caused strong defects in SDS-resistance, whereas the other substitutions had minor effects, which may relate to a partial interference with TatA binding or other effects (Fig. 4A). Assessment of Tat complex formation with the Bpa exchanges in TatC indicated that TatC(N139Bpa) strongly interfered with the formation of TC1, and a double band in the region of TC3 indicated accumulating, possibly kinetically trapped higher molecular weight assemblies (marked with a circle in Fig. 4B), which likely caused the effect on activity. In contrast, TatC(V167Bpa) and TatC(F213Bpa) exchanges had no major effect on Tat complex formations, and the observed effects on activity may have been caused by influences on TatA interactions with TatC helices IV (in case of V167Bpa) and VI (in case of F213Bpa). Note that V167 and F213 are reported to be in very close contact to TatA^30,32^, and the more bulky Bpa exchanges may therefore reduce TatA interactions with helices IV and VI that are important for activity.

Further, our data indicated that Bpa exchanges at TatC positions M205, P210, D211, and F213 selectively reduced the abundance of TC2, whereas TC1 remained well-detectable. All these exchanges are in the ‘polar cluster’ site, suggesting that TC2 formation and ‘polar cluster’ interactions are interrelated.

### The detected TatA × TatC cross-links do not change upon substrate-binding

**Figure 5:**
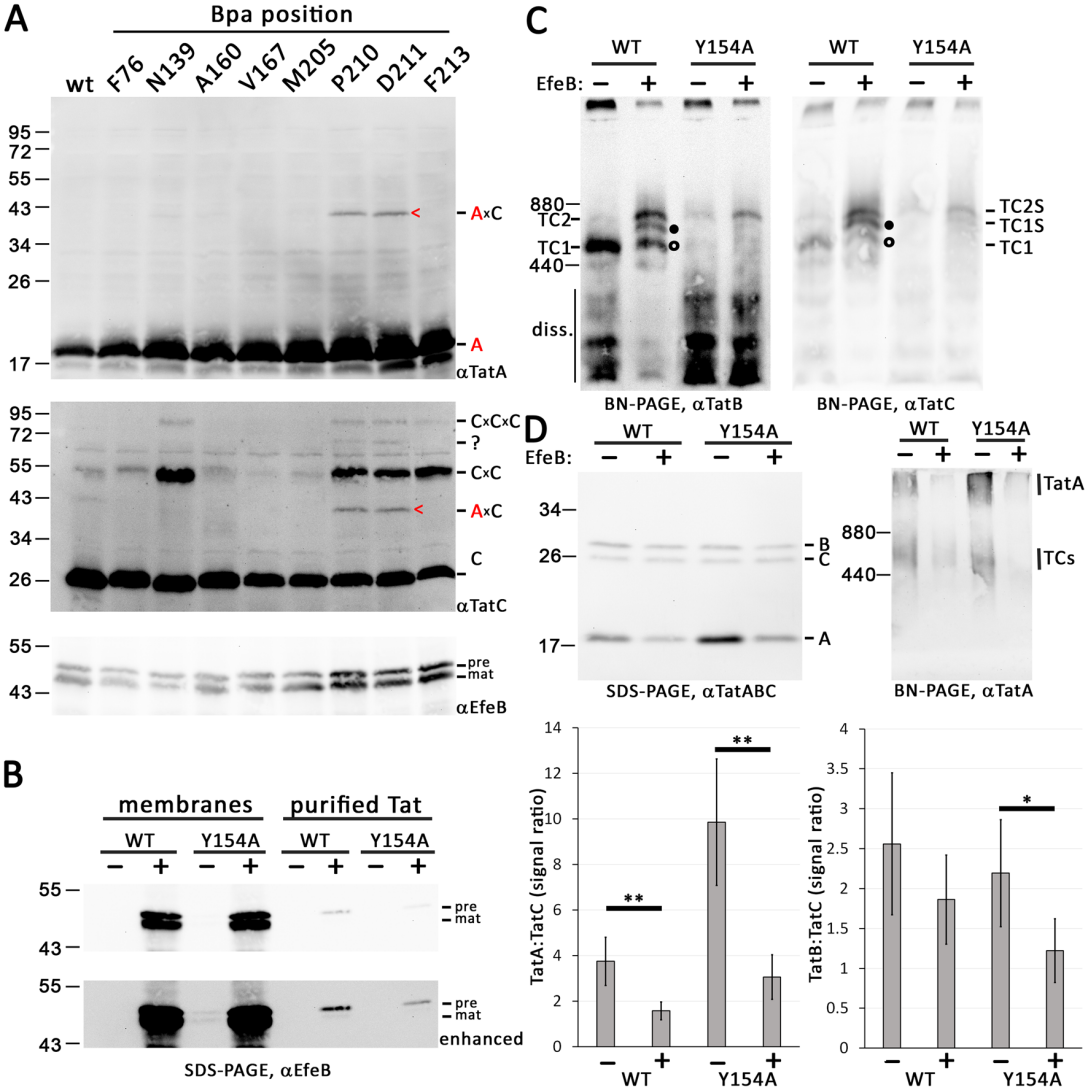
Tat-substrate saturation does not influence the Bpa cross-links to TatA but results in marked reduction of TatA co-purification. (**A**) SDS-PAGE/Western-blot analysis of cross-links with Bpa placed at indicated positions in TatC with strains overproducing EfeB (upper two blots), and detection of EfeB precursor (pre) and mature (mat) protein in the same samples. Note that the mature form of EfeB in the membrane fraction is due to some transport-unrelated signal peptide degradation. Mature EfeB apparently has some affinity to membranes, most likely due to its requirement to assemble its heme cofactor at the membrane^34^. Cross-link positions are indicated on the right. TatA × TatC cross-links are assigned based on coincident positions of the bands in TatA and TatB blots. A cross-link with an unknown partner is indicated by a question mark. (**B**) SDS-PAGE/Western blot detection of EfeB in solubilized membranes (left four lanes) and purified Tat systems (right four lanes) of strains containing wild type (WT) or TatABC(Y154A) Tat systems, each without (−) or with (+) EfeB, as indicated. Note that only precursor of EfeB is co-purified with Tat systems, whereas precursor as well as mature EfeB is present in washed membranes. (**C**) BN-PAGE analysis of Tat complexes in solubilized membranes formed by the wild type and by TatABC(Y154A) Tat systems in the absence (−) or presence (+) of recombinantly overproduced EfeB (TatB and TatC blots). Marker positions and positions of TC1 and TC2 and the substrate-bound derivatives TC1S and TC2S are indicated on the sides. The Tat complexes that show altered TatB:TatC ratios are indicated by open circles (TC1) and filled circles (TC1S). **(D) **The content of co-purified TatA is reduced in response to substrate-binding. Analyses were done after Tat complex purifications. SDS-PAGE/Western blot detection of TatABC and BN-PAGE/Western blot detection of TatA in wild type and TatABC(Y154A) systems without (−) or with (+) recombinant EfeB production. The diagrams below show a quantification of the TatA:TatC and TatB:TatC signal ratios (error bars indicate standard deviations calculated from four replicates; *t*-test significance results are indicated above the corresponding columns: **p ≤ 0.01; *p ≤ 0.05).

As saturation of Tat systems by recombinant production of transported Tat substrates has been reported to enhance the association of TatA with TatC^17,18^, and as active transport has been reported to enhance the association of TatA with the third binding site at TMH IV^30^, we also examined the effect of recombinant substrate production on the cross-links (Fig. 5A). However, when we overproduced the Tat substrate EfeB, a periplasmic iron oxidase formerly known as YcdB^34,35^, we did not detect other TatA × TatC cross-links than the above-mentioned cross-links with Bpa at positions P210 and D211 in TatC (compare with Fig. 3A). EfeB could be purified together with the Tat systems via affinity chromatography (H_6_-tagged TatC), and the Tat systems selectively interacted with the signal peptide-containing precursor of EfeB, indicating that the twin-arginine signal peptide interacted with the binding site at TatC (Fig. 5B). As expected, the known substrate-bound complexes TC1S and TC2S were formed, as detected by BN-PAGE/Western blotting^8^ (Fig. 5C). We went one step further and analyzed the substrate-bound complexes of the TatABC(Y154A) system that has no detectable TC1. The EfeB precursor also co-purified with TatABC(Y154A), but the amount of EfeB was reduced in comparison to the wild type Tat system (Fig. 5B). BN-PAGE/Western blotting analyses showed that, as expected, TC2S was the predominant substrate-binding induced complex with TatABC(Y154A), demonstrating that TC2S is indeed generated by substrate-binding to TC2 (Fig. 5C). Although TC2S was the predominant substrate-bound complex with TatABC(Y154A), it was lower abundant than in case of wild type Tat systems, which explains the lower amount of co-purified EfeB as detected in Fig. 5B.

When the TatA:TatC and TatB:TatC signal intensity ratios were determined, we noted that Tat substrate-binding caused a marked decrease of co-purified TatA, and to a lesser extent also of TatB (Fig. 5D). BN-PAGE analyses showed that TatA signals decreased in the Tat complex region as well as in the region of the co-purified high molecular weight TatA assemblies (Fig. 5D). As expected, the TC2-stabilizing TatABC(Y154A) system co-purified with more TatA than the wild type system, but the substrate-induced reduction of co-purified TatA (and TatB) was observed in both cases.

In conclusion, the recombinant production of a Tat-complex-binding Tat substrate—although not changing the cross-links—altered the stability of TatA–TatC interactions, which became less resistant against solubilization and purification.

## Discussion

TatA is required for Tat transport and somehow enables the membrane passage after Tat substrates are recognized by TatC^17–20^. So far, three TatBC-containing complexes could be differentiated by BN-PAGE, two of which, TC1 and TC2, can be readily detected with wild type Tat systems^8^. Upon substrate-binding, TC1 and TC2 are converted to TC1S and TC2S, respectively^8,10^. Our data now show that TC2 recruits more TatA than TC1 does, but in BN-PAGE analyses, most of the co-purified TatA migrates in very large associations with little or no TatBC content (Fig. 1). In addition to these large TatA clusters, some TatA is detected in rather diffuse bands in the TC1/TC2 region, indicating that a small portion of the TatA remained associated with TatBC and resisted BN-PAGE conditions, with partial and variable occupancy of TatA binding sites likely causing the diffuse band, while the predominant and more defined TC1 and TC2 bands are TatBC “core” complexes from which TatA is stripped off. Consequently, no co-purified TatA was detectable in these sharp TC1 and TC2 bands. The difference in migration behavior of TC1 and TC2 therefore does not reflect an association of TatA but rather distinct states or conformations of the TatBC core, and these distinct states differ in their TatA affinities (Fig. 2). The maximum number of direct TatA interaction sites in TatBC core complexes depends on the number of TatBC units in these complexes, which is unclear: Using fluorescence techniques, TatBC-containing complexes have been calculated to contain in average 4 TatBC units^36^. Disassembly bands that are detectable by BN-PAGE of solubilized crude membranes are suggestive for 3–6 TatBC-containing units^10^, which can be caused by dissociation of either TatB, TatC or TatBC modules. In any case, there are multiple TatA binding sites in TatBC-containing complexes, and each TatBC unit can be expected to provide at least one TatA binding site, and a low and partial occupancy of these sites explains best the diffuse BN-PAGE TatA signals. Our study now indicates that TC2 has a higher affinity for TatA, and because of the requirement of TatA for the membrane passage, the higher affinity for TatA likely relates to an advanced state of TC2 in the translocation pathway.

Most co-purified TatA is detected by BN-PAGE in very large associations, and these contain only little or no TatBC (Fig. 1). It is known since long that TatA can form large homooligomeric or nearly homooligomeric assemblies in *E. coli*^16,37,38^, but also in all other tested systems, such as *B. subtilis*^39^ or plant plastids^40^. Our results fully agree with recent biochemical data that suggested that such large homooligomeric TatA clusters remain intact even after dissociation from TatBC^41^, and they also agree with recent electron microscopical detections of large metallothionein-tagged TatA clusters at wild type expression level^21^. Our data support the view that such larger TatA assemblies interact with the ‘polar cluster’ site of TatC, as only to this ‘polar cluster’ TatA interactions could be detected (Fig. 3), and no significant changes of interactions were caused by in vivo cross-linking in response to recombinant production of Tat substrates (Fig. 5). Since, in comparison to TC1, more TatA co-purifies with TC2, TC2 must have an increased affinity for TatA, although the detected interaction site appears to be the same, suggesting that conformational transitions rather than occupancy of additional binding sites increase the affinity. A conformational transition is also supported by the fact that TC1 and TC2 migrate differently in BN-PAGE, although their prominent bands actually lack TatA that largely dissociates under BN-PAGE conditions (see above). Note that our cross-linking did not monitor any exchanges of TatB and TatA at the binding sites at TatC helices V and VI, as our Bpa-based method did not resolve these nearby interaction sites, and therefore our data do not contradict the conclusions made by Habersetzer et al.^12^. Moreover, as we observed a reduction of TatB in cases of increased TatA levels, our analyses fully agree with and even support the idea that TatA can compete with TatB for the same binding sites (Fig. 2B). We did not observe a TatB cross-link to TatC(M205Bpa) in TMH V, although others have observed this^31^. In that study, a much stronger, IPTG inducible T7-promoter-dependent expression system was used for the production of the Tat components, and cross-linking was done in vitro after preparation of inverted membrane vesicles. It might well be, that under such experimental conditions, TatB or TatA can become detectable as a cross-link to TatC(M205Bpa). Our in vivo cross-linking was done with a constitutively produced and physiologically functional Tat system. If TatC(M205Bpa) would disrupt a functionally important interaction of TatC with TatB or TatA, we would have expected a more severe effect on activity, which was not observed (Fig. 3B). We therefore do not think that such interactions are significantly affected, and the reason for the absence of the cross-links to TatC(M205Bpa) may simply be a low cross-linking efficiency and therefore a difficult detection without strong overproduction. However, since we could monitor the TatA interaction to the ‘polar cluster’ site by TatC(P210Bpa) and TatC(D211Bpa), and since the aspects of differential binding of TatB or TatA to helices V and VI have been extensively addressed elsewhere and are in full agreement with our data^12,32^, we did not address this point again in this study.

Important additional results are related to effects that substrate-binding has on Tat systems, namely (i) that TC2S contains more TatB than TC1S (Fig. 5C), and that (ii) substrate-binding reduces the amount of TatA that can be co-purified with Tat systems (Fig. 5D). The ratio of TatB:TatC in the Tat complexes is obviously not the same (Fig. 5C; compare TC1, TC1S, and TC2S band intensities in TatB and TatC blots for lanes wt + EfeB). Also, the change in TatB:TatC ratio in Tat systems with TatC mutations that stabilized TC1 or TC2 (Fig. 2) supports this view: There is no strict 1:1 TatB:TatC ratio, even in the core TatBC complexes. This 1:1 ratio originally has been postulated based on TatB–TatC fusion experiments, and quantification of TatABC component signals in elution fractions of Tat complex purifications after ^35^S methionine-labelling^11^. However, the TatB:TatC ratio varied in these original measurements, with TatB usually showing a slight excess over TatC. On transcriptional level, TatB is produced in 2.5× excess over TatC^42^, and the fact that TatA can occupy TatB binding sites and vice versa^12^ can explain why there does not need to be a fixed 1:1 ratio in all TatBC-containing complexes.

Another important aspect is the significant reduction in co-purified TatA upon recombinant production of the Tat substrate EfeB that binds the Tat system and is co-purified (Fig. 5D). This effect fully agrees with the previous observation of increased TatA co-purification in affinity chromatographies with tagged TatC when the Tat substrate binding site has been mutated^10^. As TC2 associates with more TatA than TC1 (Fig. 2), and as TatA is required for the membrane passage, it is clear that many TatA protomers are associated when TC2S is formed. However, as substrate-binding results in significantly less co-purification of TatA, the substrate must cause changes that destabilize the TatA–TatC interaction.

**Figure 6:**
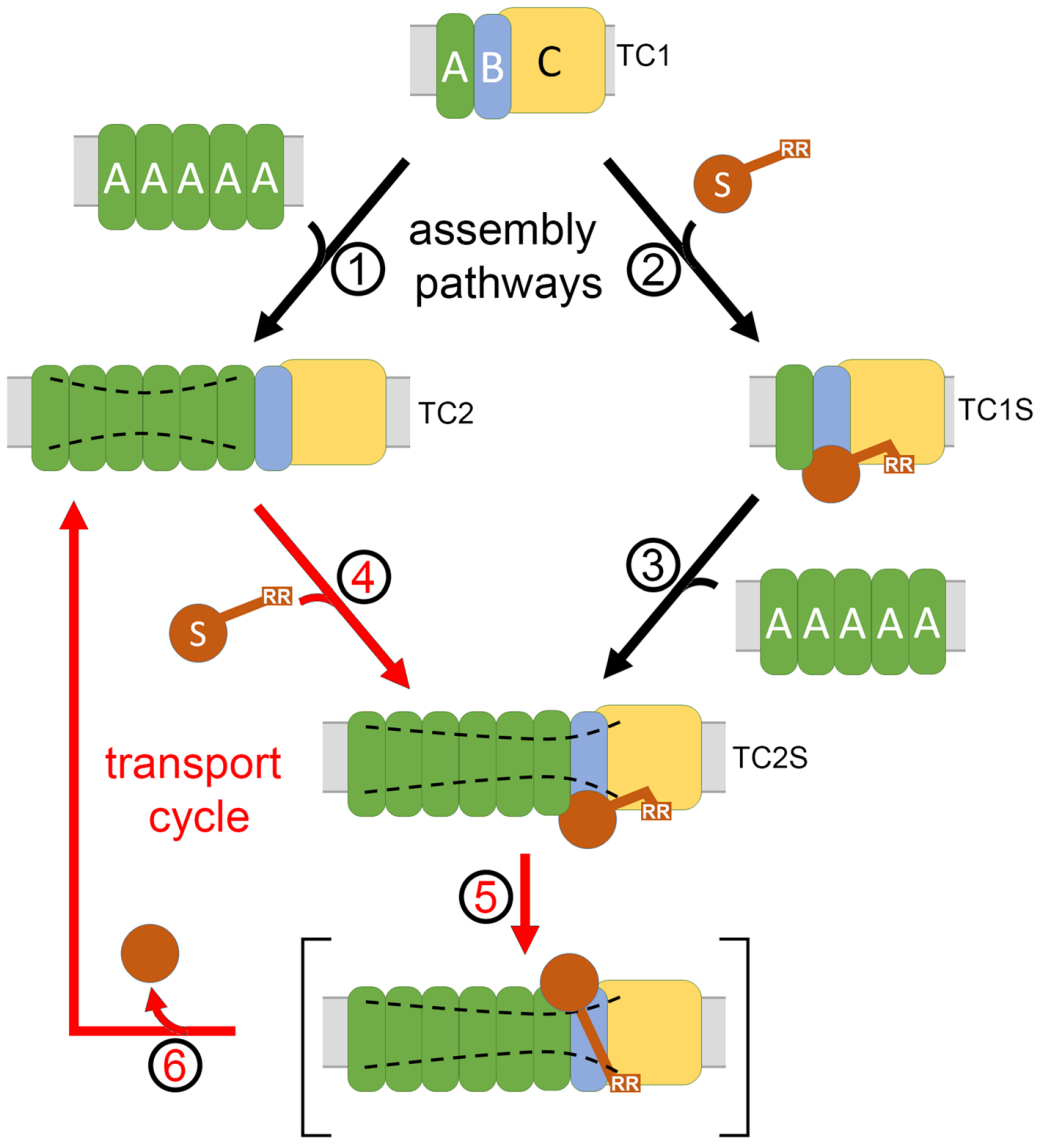
Model for Tat translocon assembly and translocation pathways, taking the new insights of this study into account. TC1 can assemble TatA and Tat substrate in any order (steps 1 → 4 or 2 → 3), finally resulting in the substrate-bound TC2S complex^10^, in which TatA is recruited to TatBC complexes as a large oligomer (this study, Fig. 1). TatA per se can already destabilize membranes, indicated by curved dashed lines in TC2 and TC2S^19,20^. Binding of the substrate’s signal peptide to TatC likely causes conformational changes as it induces further membrane-destabilization^19^. Bound substrate likely extends the membrane-destabilization into the TatBC region, as it affects the TatA–TatBC interaction (this study, Fig. 5), and this likely permits translocation (step 5). The TatA–TatC interaction site is not significantly altered by substrate-binding (this study, Figs. 3 and 5), indicating that there are no major rearrangements that involve the laterally associated TatA. Upon transport, the substrate is readily released, resetting the system to the TC2 complex (step 6). The simplest translocation mechanism would be a cyclic pathway in which the large TatA clusters remain bound to TC2. *S* substrate, *A* TatA, *B* TatB, *C* TatC. The position of the RR-motif near the N-terminus of the signal peptide is schematically indicated.

Early in Tat research, diverse mechanistic models have been proposed for the mechanism of TatA-dependent membrane permeabilization, ranging from formation of gated aqueous pores and flexible iris-like rings^43^ to local membrane-weakening^22^. After manyfold structural insights and biochemical analyses, it is now clear that TatA is locally thinning the membrane, resulting in membrane destabilization that is caused by conserved characteristics of TatA, and it appears that this membrane-thinning is crucial for TatA function, whereas other suggested transport mechanisms could not be experimentally supported^6,19–21,44^. Whatever the oligomeric structure is that is formed by TatA, it is clear that proteins must be transported through a lipid-rich environment^24^. MD simulations suggest that multiple TatA protomers stably form two-row linear associations with their trans-membrane helices, and the membrane is significantly thinned in their central region^21^. Substrate-binding enhances membrane destabilization by TatA^19^, and large TatA clusters change their mobility in destabilized membranes^45^. We observed that substrate-binding strongly affects the TC2 interaction (Fig. 5), suggesting that the membrane-thinning by TatA is extended to its TatBC interaction site near TatC helices 5 and 6 upon substrate-binding. All these aspects can be incorporated in an updated mechanistic model for Tat translocon assembly and translocation (Fig. 6). In this model, the formation of TC2 or TC2S from TC1 can be seen as assembly pathways (black arrows). Once the large TatA clusters are recruited, substrate-binding, transport, substrate-release, and resetting to TC2 can be repeated in cycles, which simplifies the pathway (red arrows). In agreement with this, we found that TC2S is the dominant substrate-bound Tat complex (Fig. 5C and Ref.^10^). Note that the PMF is known to be required for transport^46,47^, but we currently do not know at which step the PMF is required and to what extent TatA needs to dissociate from TatBC for release of the transported substrate, or whether it needs to dissociate at all. These are interesting aspects that remain to be clarified in future studies.

## Methods

### Strains and growth conditions

*Escherichia coli* XL1-Blue MRF’ tet (Agilent) was used for cloning. *E. coli* DADE D6 ara^R^^48^ was used for purification and cross-linking experiments. *E. coli* was cultivated aerobically at 37 °C in LB medium (1% (w/v) tryptone, 1% (w/v) NaCl, 0.5% (w/v) yeast extract) with the appropriate antibiotics (25 µg/ml chloramphenicol, 12.5 µg/ml tetracycline, 100 µg/ml ampicillin). For purifications, 1 l LB medium was inoculated to an OD_600_ of 0.1 with an overnight culture. For *efeB* overexpression, 0.1% (w/v) rhamnose was added at OD_600_ of 0.6. Cultures were harvested after reaching an OD_600_ of 1, or 3 h after induction in case of rhamnose induced cultures, and cell densities were normalized to an OD_600_ of 1. Cells were harvested by centrifugation (7000×*g*, 4 °C, 15 min), washed with 50 ml PBS, and centrifuged again. For the incorporation of Bpa, 100 µM Bpa and 100 µM arabinose were added to 100 ml LB before inoculation with 4 ml overnight culture. For cross-linking, cultures were incubated for 5 h at 37 °C and irradiated with UV light at 365 nm for 30 min at room temperature before normalizing to an OD_600_ of 1. Cell pellets were frozen in liquid nitrogen and stored at − 20 °C before further use. For SDS resistance assays, OD_600_ values of cultures grown in liquid LB ± 4% SDS were compared after 5 h growth.

### Genetic methods and plasmids

All primers for *tatC* codon exchanges are listed in Table 1. The plasmids used for expression of *tatABC* (with wild type or mutated *tatC* genes) were pABS-*tatABC-H6* in case of purifications and pDE-*tatABC-H6* for cross-linking experiments^27^. All constructs were confirmed by sequencing. For overexpression of *efeB*, the plasmid pBW-*efeB-strep* was used^34^. pEVOL-Bpa-tet, used for the incorporation of Bpa at introduced amber stop codons, was donated by Peter G. Schultz^49^.

**Table 1:** Primers used in this study^1^.

QuikChange mutagenesis primers^2^	Sequence (5′ > 3′)
*TatC-F118A-F-jb*	CCAGCTCTCTGCTGGCCTATATCGGCATGGC
*TatC-Y154A-F-jb*	CACCGACATCGCCAGCGCCTTAAGCTTCGTTATG
*TatC-L189A-F-jb*	CCTCGCCAGAAGACGCCCGCAAAAAACGCCCG
*TatC-F76Bpa-F-MW*	CGATCAAGCTGACCTAGATGGTGTCGCTGATTC
*TatC-N139Bpa-F-MW*	CATTTGGCTTCCTTGCCTAGACCGCGCCGGAAGGGG
*TatC-A160Bpa-F-MW*	GCTATTTAAGCTTCGTTATGTAGCTGTTTATGGCGTTTGGTG
*TatC-V167Bpa-F-MW*	CTGTTTATGGCGTTTGGTTAGTCCTTTGAAGTGCCGGTAG
*TatC-M205Bpa-F-MW*	CATTCGTTGTCGGGTAGTTGCTGACGCCG
*TatC-P210Bpa-F-MW*	GGATGTTGCTGACGCCGTAGGATGTCTTCTCGCAAAC
*TatC-D211Bpa-F-MW*	GATGTTGCTGACGCCGCCGTAGGTCTTCTCGCAAACGCTG
*TatC-F213Bpa-F-MW*	CCGCCGGATGTCTAGTCGCAAACGCTG

^1^Primer sequences are given in 5′–3′ direction. ^2^Corresponding reverse primers covered the same sequence.

### Biochemical methods

Blue Native PAGE was performed as described previously^8^. Membrane proteins were solubilized with 2% (w/v) digitonin and purified by affinity chromatography using 0.5 ml Protino Ni-NTA agarose (Macherey-Nagel) gravity flow columns. The columns were equilibrated with 10 column volumes of 50 mM Bis–Tris, 20% (w/v) sucrose, 10 mM MgCl_2_, 0.1% (w/v) digitonin, pH 7.0. Before applying to the column, the solubilized membranes were diluted fourfold with solubilization buffer to reduce the digitonin concentration to approximately 0.5% (w/v). After loading the solubilized membranes, the columns were washed with 10 column volumes of wash buffer (50 mM Bis–Tris pH 7.0, 50 mM MgCl_2_, 0.1% (w/v) digitonin, 20% sucrose, 500 mM imidazole). Elution was performed with elution buffer (50 mM Bis–Tris, 10 mM MgCl_2_, 20% (w/v) sucrose, 0.1% (w/v) digitonin, 200 mM imidazole, pH 7.0). Elution fractions of 500 µl each were collected and the elution fraction that contained most eluted protein (E2) was used for further analyses.

SDS-PAGE and silver staining were carried out by standard protocols^50,51^. Samples were not heated to prevent aggregation of TatC. The assignment of the detected protein bands was confirmed by Western blotting (Supplementary Fig. S1). Proteins were blotted on nitrocellulose membranes using semi-dry Western blotting. After blotting, nitrocellulose membranes were blocked with 5% (w/v) skim milk in PBS for 1 h or overnight and washed 3 times with PBS for 5 min. Blots were developed using primary antibodies against TatA, TatB, TatC, or EfeB^8^. For the determination of TatA:TatC and TatB:TatC signal ratios, TatA, TatB and TatC antibodies were mixed in an 1:1:1 ratio. Goat-anti-rabbit IgG HRP conjugate (Advansta) was used as secondary antibody. For detection, the ECL system (GE Healthcare) was used. Quantification of Western blot bands was performed with Image lab software (Bio-Rad). After manual lane annotation and background subtraction, the TatA:TatC and TatB:TatC signal ratios were analyzed by setting the TatC band as reference signal.

### Supplementary Information

Supplementary Figures.

## Data Availability

The authors declare that the data supporting the findings of this study are available within the paper and its Supplementary Information file. Should any raw data files be needed in another format they are available from the corresponding author upon reasonable request.

## Abbreviations

BN-PAGE: Blue-native polyacrylamide gel electrophoresis
Bpa: *p*-Benzoyl-phenylalanine
IMAC: Immobilized metal affinity chromatography
NMR: Nuclear magnetic resonance
SDS: Sodium dodecyl sulfate
Tat: Twin-arginine translocation
TC: Tat complex
TMH: Transmembrane helix
