# Supplementary material for: A larger TatBC complex associates with TatA clusters for transport of folded proteins across the bacterial cytoplasmic membrane
Source: Sci Rep. 2024 Jun 14;14:13754. doi: 10.1038/s41598-024-64547-x (PMC11178869; doi:10.1038/s41598-024-64547-x)
Supplement: Supplementary file 1 — Supplementary Figures. [file 41598_2024_64547_MOESM1_ESM.pdf]

A larger TatBC complex associates with TatA clusters for transport of folded proteins across the bacterial cytoplasmic membrane

Supplementary Information

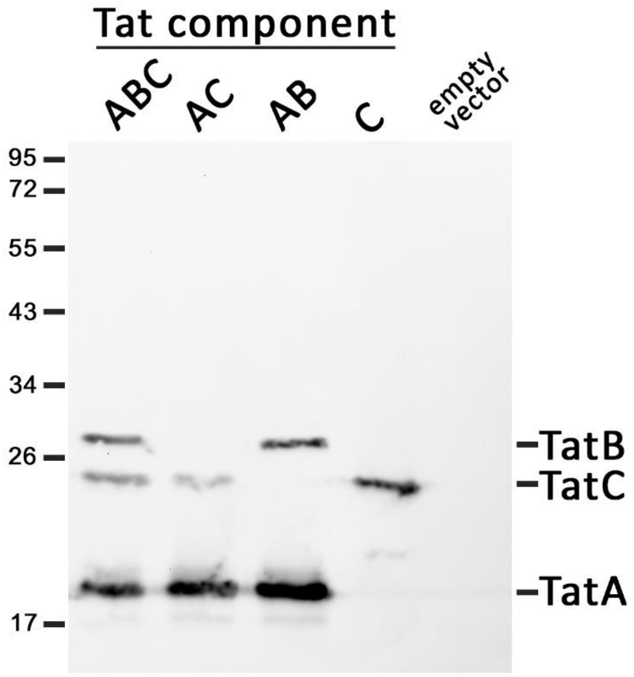

Fig. S1: The mixture of antibodies used in this study specifically recognizes TatA, TatB, and TatC. SDS-PAGE/Western blot analysis of membrane preparations of *E. coli* DADE D6 ara^R, containing the plasmids pABS-*tatABC* [1], pABS-*tatAC*, pABS-*tatAB*, pABS-*tatC*, or pABS (empty vector, [1]) for the P_{tatA}-dependent constitutive production of the indicated Tat components. For pABS-*tatC*, SnaBI sites were introduced in pABS-*tatABC* by QuikChange mutagenesis behind the start codon of *tatA* and in frame in the start codon of *tatC*, and *tatAB* were deleted by SnaBI digest and religation. For pABS-*tatAB*, SnaBI sites were introduced in the start and stop codons of *tatC*, and *tatC* was deleted by SnaBI digest and religation. pABS-*tatAC* was generated by mutation of the *tatB* start codon (cgtgtt > tacgta, a SnaBI restriction site). Blots were developed with a mixture of antibodies recognizing TatA, TatB, or TatC.

[1] Berthelmann F. and Brüser T. (2004). Localization of the Tat translocon components in *Escherichia coli*. FEBS Lett 569: 82–88. doi: 10.1016/j.febslet.2004.05.054

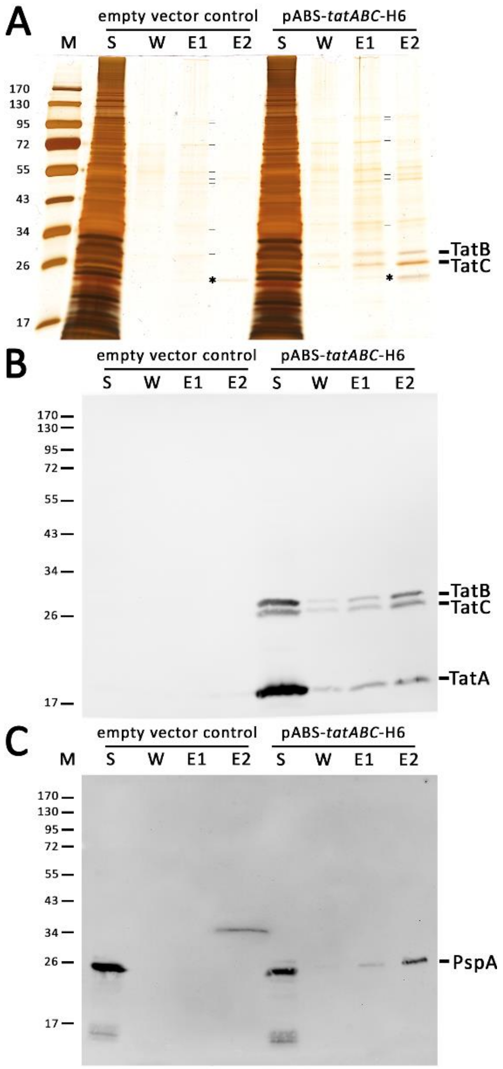

Fig. S2: Detection of Tat-unrelated inpurities and of PspA after affinity purification of TatC-H6. (A) SDS-PAGE/silver staining analysis of a mock purification from the empty vector control strain, in comparison with a purification from a strain containing the constitutive expression vector pABC-*tatABC*-H6. Analyzed are solubilized membranes (S), the last wash fraction (W), and elution fractions 1 and 2 (E1, E2). M, marker proteins. Molecular masses of the marker proteins are indicated on the left (in kDa). Note that most silver-stainable bands other than TatB and TatC are also detected in the control elution, although the amount of inpurities varied between purifications. Positions of prominent Tat-unrelated bands are indicated by hyphens and an asterisk (the most prominent band). (B) Detection of TatA, TatB, and TatC by Western-blot in the fractions analyzed in (A), using the mixture of specific antibodies. (C) Detection of PspA by Western-blot in the same fractions analyzed in (A) and (B), using a PspA-specific antibody.

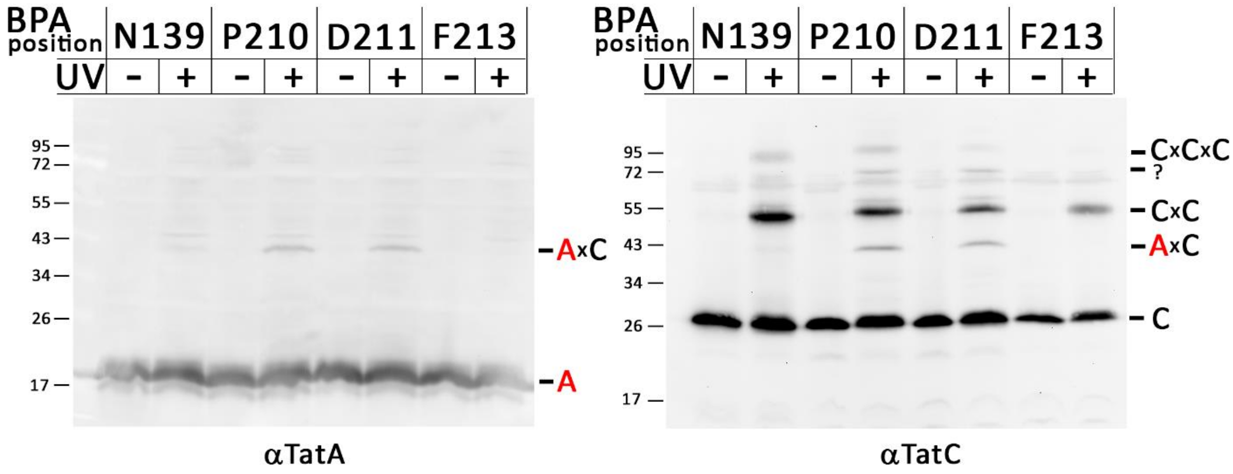

Fig. S3: The observed BPA cross-links are UV-dependent. Confirmation of UV-dependence of the cross-links detected in the experiments shown in Fig. 3 and Fig. 5.
